# Draft Genome Sequence and Annotation of the Insect Pathogenic Bacterium Xenorhabdus nematophila Strain C2-3, Isolated from Nematode *Steinernema carpocapsae* in the Republic of Korea

**DOI:** 10.1128/genomeA.01521-14

**Published:** 2015-02-12

**Authors:** Sung-Jun Hong, Ihsan Ullah, Gun-Seok Park, Byung Kwon Jung, JungBae Choi, Abdur Rahim Khan, Min-Chul Kim, Jae-Ho Shin

**Affiliations:** School of Applied Biosciences, Kyungpook National University, Daegu, Republic of Korea

## Abstract

Xenorhabdus nematophila strain C2-3, which belongs to the family Enterobacteriaceae, was isolated from entomopathogenic nematodes collected in the Republic of Korea. Herein, we report a 4.38-Mbp draft genome sequence of X. nematophila strain C2-3, with a 43.6% G+C content. The RAST annotation analysis revealed 4,994 protein-coding sequences in the draft genome.

## GENOME ANNOUNCEMENT

Xenorhabdus spp. are *Enterobacteriaceae*, symbiotic bacteria associated with soil nematodes such as *Steinernematidae* and *Heterorhabditidae* spp. (1). Entomopathogenic bacteria symbiotically associated with nematodes are of great interest as biocontrol agents against insect pests (2, 3). Xenorhabdus spp. are virulent pathogens synthesizing proteins and other secondary metabolites, such as benzylideneacetone, iodinine, phenethylamides, xenorhabdins, xenorxides, and xenocoumacins, involved in pathogenicity against a wide range of insects (4). Previous studies have revealed numerous secondary metabolitic activities, e.g., antibacterial activity, antifungal activity, insecticidal activity, and cytotoxicity, investigated in other Xenorhabdus strains (5).

In the present study, bacterial strain C2-3 was isolated from nematodes collected in the Republic of Korea. The strain was identified through 16S rRNA sequence comparison, which revealed 99.8% sequence similarity with Xenorhabdus nematophila ATCC 19061, and it was therefore named Xenorhabdus nematophila strain C2-3. Moreover, the identification was confirmed via average nucleotide identity (ANI) values (6). The whole-genome sequence of the C2-3 strain showed 98.9%, 81.9%, 80.8%, and 79.7% ANI values for X. nematophila ATCC 19061 (1), X. szentirmaii DSM16338 (7), and X. bovienii SS-2004 (1), respectively.

The newly identified strain C2-3 was subjected to draft genome sequencing to investigate the presence of insecticidal toxins, secondary metabolites, and antimicrobial compounds. The genomic DNA from X. nematophila C2-3 was extracted using a QIAamp DNA minikit (Qiagen, Hilden, Germany), and the whole genome was sequenced with the Ion Torrent PGM sequencer (Thermo Scientific, Bremen, Germany), using the 316 v2 chip sequencing protocol. A total of 3,310,612 reads were generated, with a mean length 272 bp. The draft genome sequence was assembled *de novo* using MIRA assembler version 4.0, which generated 284 contigs (500 bp or more), with an *N*_50_ contig length of 48,919 bp. The draft genome sequence consists of 4,386,383 bp, with 60-fold genome coverage having approximately 43.6% G+C content.

Subsequent to the assembly, the contigs were submitted to the RAST annotation server (http://rast.nmpdr.org) for subsystem classification and functional annotation (8). The annotation results revealed 4,994 predicted coding sequences, including 67 tRNAs, 32 rRNAs, and 4 noncoding RNAs. In addition, there were two complexes, *xpt*A1/*xpt*B1/*xpt*C1 and *xpt*A2/*xpt*B1/*xpt*C1 (9, 10). These toxin complexes have 47% to 53% amino acid sequence similarity with that of the toxin complex (TC) protein of P. luminescens (11). Moreover, the *xpt*A1 gene encoding central insecticidal toxin, as well as *xpt*B1 and *xpt*C1 genes encoding toxicity enhancer proteins (10), were clustered.

In addition, an antimicrobial gene cluster identified as *xcn*A-N and related to xenocoumacin production (12) was also revealed. Based on the annotation results, we presume that the genome sequence of X. nematophila strain C2-3 will lead to the discovery of useful genes and gene products for environmentally friendly agriculture applications.

### Nucleotide sequence accession numbers.

This whole-genome shotgun project has been deposited at DDBJ/EMBL/GenBank under the accession no. JRJV00000000. The version described in this paper is version JRJV01000000.
